# Cocaine-Induced Microglial Impairment and Its Rehabilitation by PLX-PAD Cell Therapy

**DOI:** 10.3390/ijms26010234

**Published:** 2024-12-30

**Authors:** Hilla Pe’er-Nissan, Pnina Shirel Itzhak, Iris Gispan, Racheli Ofir, Gal Yadid

**Affiliations:** 1 Neuropharmacology Laboratory, The Mina & Everard Goodman Faculty of Life Sciences, Bar-Ilan University, Ramat Gan 5290002, Israel; peer.hilla@gmail.com (H.P.-N.); pnina80206@gmail.com (P.S.I.); iris.gispan@biu.ac.il (I.G.); 2 The Leslie and Susan Gonda (Goldschmied) Multidisciplinary Brain Research Center, Bar-Ilan University, Ramat Gan 5290002, Israel; 3 Pluristem Therapeutics Inc., Haifa 3508409, Israel; racheli@pluristem.com

**Keywords:** addiction, microglia, Iba-1, cocaine, drug self-administration, mesenchymal stem cell, cell therapy, PLX-PAD, intranasal administration

## Abstract

Chronic cocaine use triggers inflammatory and oxidative processes in the central nervous system, resulting in impaired microglia. Mesenchymal stem cells, known for their immunomodulatory properties, have shown promise in reducing inflammation and enhancing neuronal survival. The study employed the cocaine self-administration model, focusing on ionized calcium-binding adaptor protein 1 (Iba-1) and cell morphology as markers for microglial impairment and PLX-PAD cells as a treatment for attenuating cocaine craving. The results revealed an addiction-stage and region-specific impairment in microglia following chronic cocaine exposure, with deficits observed in the Nucleus Accumbens (NAc) during the maintenance stage and in both the NAc and Dentate Gyrus (DG) during the extinction and reinstatement stages. Furthermore, PLX-PAD cell therapy demonstrated a significant reduction in cocaine craving and seeking behavior, interestingly accompanied by the prevention of Iba-1 level decrease and restoration of microglial activity in the NAc and DG. These findings highlight the unique role of microglia in modulating cocaine addiction behaviors through their influence on synaptic plasticity and neuronal remodeling associated with memory formation. They also suggest that PLX-PAD therapy may mitigate the detrimental effects of chronic cocaine exposure on microglia, underscoring the importance of incorporating microglia in comprehensive addiction rehabilitation strategies.

## 1. Introduction

Microglia, the resident immune cells in the central nervous system (CNS) [1], provide the first line of defense and play a critical role in maintaining homeostasis [1,2] by adopting various functional states, including surveillance and pro-inflammatory and anti-inflammatory states, depending on the context [3]. Microglia are involved in essential brain functions, such as detecting critical changes in neuronal activity and responding rapidly to minor changes in the brain [4]. In the adult brain, microglia influence synapse remodeling, learning and memory formation, and the maturation of new neurons [5,6] by phagocytosing redundant structures, creating space for integrating new neurons [7,8,9], and secreting survival and differentiation factors for neural progenitor cells [10,11]. Additionally, microglia promote the formation of learning and memory-dependent synapses through the secretion of brain-derived neurotrophic factor (BDNF) [11]. Microglia also regulate cognitive activity and synaptic plasticity by mediating synaptic pruning, directly affecting neuronal activity in the hippocampus [12,13,14]. Disruptions in synaptic pruning, as seen in conditions like depression [15,16] and Alzheimer’s disease, are associated with altered neuronal connectivity [5] and social abnormalities [7].

Growing evidence correlates the role of the immune system with the development of substance use disorder. Addictive substances, such as cocaine, ethanol, and opioids, activate the immune system in both rodent and human CNSs [17]. Cocaine directly binds to microglial receptors, triggering the release of pro-inflammatory cytokines like IL-1β, which increases synaptic dopamine levels and pleasure sensation [18,19]. In addition, cocaine interacts with the dopamine transporter, further elevating dopamine levels and stimulating the reward pathway [20]. These processes also trigger inflammatory and oxidative responses that impair microglial function [21] and reduce neurogenesis in the Dentate Gyrus (DG) [22,23]. The ablation of microglia has been shown to reduce neuroblast survival in the adult hippocampus [22].

The sustained and unregulated activation of microglia due to the consequence of repeated and prolonged exposure to cocaine during addiction induces excessive and persistent oxidative stress and neuroinflammation, leading to continuous neuronal deterioration and progressive neurodegeneration [24]. While moderate activation of microglia is crucial for an initial response to brain insult, persistent activation leads to exacerbated neuroinflammation and neuronal death. Subsequently, the microglial production of pro-inflammatory cytokines is further stimulated by specific factors released from stressed or damaged neurons. Microglial over-activation has been documented in numerous neurodegenerative conditions, like Parkinson’s disease [25], Alzheimer’s disease [26], Amyotrophic Lateral Sclerosis (ALS) [27], and psychiatry disorders such as depression [28] and drug addiction [24,29,30].

Cocaine self-administration negatively affects neurogenesis, synaptic strength, and the plasticity of existing neurons in the hippocampus, contributing to cognitive deficits and disrupting memory processes. These disruptions can evoke memories of past drug-induced seeking behavior and increase the propensity for relapse [4,6], as reduced neurogenesis has been associated with increased susceptibility to relapse and impaired cognitive performance, both of which are critical in sustaining addiction [31,32]. In addition, decreasing neurogenesis prior to drug exposure in animals increases vulnerability to drug-seeking behavior [7]. Interestingly, a recent study has shown that PLX-PAD cell therapy rehabilitates cocaine-seeking behavior by migrating to specific mesolimbic regions, improving the plasticity by the restoration of neurogenesis in the Dentate Gyrus [32].

Microglial activity is commonly studied using ionized calcium-binding adaptor protein 1 (Iba-1), a biomarker expressed in glial cells [33,34]. Iba-1 binds actin to form actin bundles, which shape the cell structure of microglia. The actin bundles support membrane ruffling, a process observed during cell migration and phagocytosis [34]. This dynamic cytoskeleton, regulated by Iba-1, allows microglia to respond to environmental changes and shape neuronal structure and plasticity [35]. Additionally, Iba-1 is involved in the regulation of intracellular calcium levels, which play a role in the activity-dependent response of microglia to stimuli [34]. During immune activation, increased Iba-1 levels cause changes in microglia, enabling them to conduct phagocytic and pro-inflammatory activities, which, if prolonged, may result in tissue damage. After the insult is eliminated, microglia typically return to a resting state, allowing tissue repair [36]. However, chronic cocaine exposure, using a dose known to impair plasticity and neurogenesis [31], can lead to excessive microglial activation, followed by impairment marked by decreased Iba-1 expression [37]. While Iba-1 serves as a useful marker, its expression in both reactive and quiescent microglia limits its ability to fully capture microglial activation [33,34], necessitating additional morphological and functional assessments.

Studies on cocaine exposure have shown varied effects on microglia activation, expressed by Iba-1 [38]. For instance, repeated cocaine injections (20 mg/kg, i.p.) over seven days increased Iba-1 levels in the cortex and striatum in mice [19,39]. However, sole passive injections with no behavioral model are not appropriate for studying addiction. In contrast, lower-dose cocaine injections (15 mg/kg, i.p. over seven days) in a conditioned place preference paradigm did not alter the estimated number of Iba-1+ cells in the nucleus accumbens (NAc) [40]. The difference in Iba-1 levels between these studies is likely due to the dose difference. Also, the conditioned place preference primarily evaluates preference and does not fully capture addiction, thus offering a limited scope in comprehensively understanding addiction.

The self-administration model provides valuable insights into microglial responses across addiction stages. This model more accurately reflects clinical evidence of addiction by mimicking human-like behavior in animals [32,41]. In this model, animals self-administer cocaine by pressing a lever [41] or nose-poking [42], leading to behavioral and neurological changes that resemble human addiction. This model is used to investigate the neural and behavioral mechanisms underlying drug addiction, including drug reinforcement, tolerance, withdrawal, and relapse, and can assist in the development of new treatments for human drug addiction [42,43]. Studies using this model have shown increased levels of Iba-1 in the NAc during the maintenance phase of addiction but decreased levels during extinction training. One study involving cocaine self-administration in mice (1 mg/kg daily 2 h sessions for three weeks) showed significant upregulation of Iba-1 intensity levels in the dorsal striatum and NAc but not the hippocampus. However, this study did not exceed the maintenance stage of addiction [36]. Another study (1 mg/kg daily for 12 days) exhibited an increased Iba-1 expression in the NAc during the maintenance phase, followed by a decrease during extinction training [44], suggesting microglial impairment. Despite these findings, the effects of cocaine on microglia in the hippocampus remain unclear, and additional studies are needed to better understand the interplay between microglia, neurogenesis, and addiction-related behaviors.

Mesenchymal stem cells (MSCs) play a crucial role in regulating microglial activity [45], reducing inflammation and oxidative stress, and enhancing neuronal survival [46,47]. Placenta-derived MSC-like cells (PLX-PAD) are FDA-approved, with immunomodulatory and regenerative properties similar to MSCs [48] and with limited proliferation and minimal differentiation capabilities [48,49], mitigating risks associated with conventional MSCs, such as tumorigenesis and concerns about post-transplantation cell fate [50]. Notably, these cells have the capability to penetrate the blood–brain barrier and migrate to addiction-related brain regions like the NAc and the DG [32]. In a rat model of cocaine addiction, PLX-PAD therapy reduced cocaine craving and seeking behavior and restored neurogenesis in the DG [31,32,51]. Considering the role of microglia in synaptic remodeling and neurogenesis, it is plausible that PLX-PAD therapy facilitates addiction recovery by rehabilitating impaired microglia.

The current study examines the effects of chronic cocaine self-administration on microglia in two brain regions: the NAc, associated with reward regulation and strengthening cocaine craving [52], and the DG, associated with altering the plasticity of cells, i.e., allowing learning and memory [53]. We hypothesized that chronic cocaine consumption would lead to microglial impairment in the NAc and DG only in the extinction and reinstatement stages, where learning and memory are critical for rehabilitation: learning the dissociation of drug consumption from reward versus forgetting the reward of drug consumption. We also hypothesized that PLX-PAD cells, due to their immunomodulatory effect, would repair the cocaine-derived microglial impairment in the extinction and reinstatement stages, enabling learning of the dissociation of drug consumption from reward in the DG and elucidating the involvement of microglia in addiction. To test these hypotheses, we employed a self-administration model and a cocaine dose known to impair neurogenesis [31,32] and evaluated the microglia through three complementary approaches: quantification of microglial cell numbers to rule out a decrease in microglia due to necrosis, analysis of Iba-1 levels, and assessment of structural changes in microglia, where increased soma surface area and decreased number of processes and branching indicate activated microglia [54]. These measures were combined with behavioral analyses across all addiction stages to elucidate the role of microglia in addiction and the potential therapeutic effects of PLX-PAD cells.

## 2. Results

We first examined the impact of chronic cocaine consumption in the maintenance stage on behavior and on Iba-1 levels in the NAc and DG of cocaine-trained rats (‘cocaine’) vs. saline-trained rats (‘Sham’) (Section 2.1). Then, we established the effect of PLX-PAD cell therapy on cocaine craving in the extinction training and reinstatement phases (Section 2.2), followed by an evaluation of behavior and Iba-1 levels of cocaine-trained rats (‘cocaine’) vs. cocaine-trained rats treated with PLX-PAD cells (‘PLX-PAD’) vs. saline (‘Sham’) (Section 2.3). Last, we examined the microglia morphology of all groups in the different addiction stages (Section 2.4).

### 2.1. Maintenance Stage—Iba-1 Levels in the NAc and DG and Behavioral Assessment

To examine the impact of chronic cocaine consumption on microglia in the maintenance phase of addiction, we used the self-administration model as described in the Methods (Section 4) with rats trained to self-administer with cocaine or saline (*n* = 8–9). We carried out a behavioral assessment of active vs. demo lever presses, and when maintenance of cocaine consumption was attained, rats were immediately sacrificed, and brain slices from the NAc and DG were collected for analysis to measure Iba-1 levels as described in the Methods section (Section 4.6).

#### 2.1.1. Cocaine-Trained Rats Discriminate Between Active and Demo Levers

**Figure 1:**
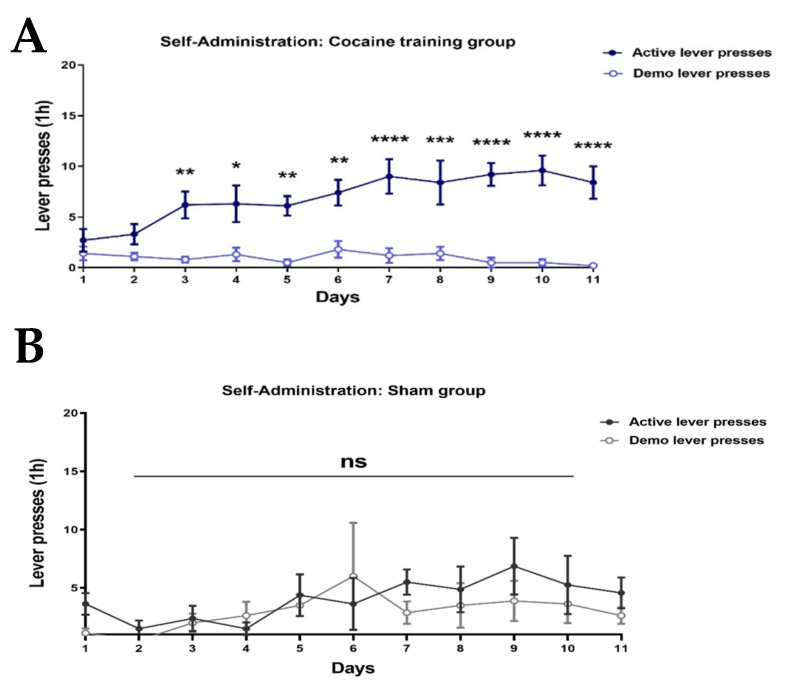
Cocaine or saline self-administration. Rats were trained to self-administer cocaine (1.5 mg/kg) (**A**) or saline (**B**) on a fixed ratio 1 schedule of reinforcement in a daily 1 h session until stable maintenance levels were attained. Cocaine-trained rats exhibited significant active and inactive (demo) lever press discrimination.. saline-trained rats (‘Sham’) did not discriminate between the active lever and the demo lever. Bonferroni’s multiple comparison post hoc; * *p* < 0.05, ** *p* < 0.01, *** *p* < 0.001, **** *p* < 0.0001, no significant effect; ns, *n* = 8–9. Overall: data show mean ± SEM.

Behavioral results indicate that saline-trained rats (‘Sham’) did not discriminate between the active lever and the demo lever (Figure 1B). Two-way ANOVA with repeated measures of active lever presses over Days 1–11 revealed no significant effect of day [F (10, 152) = 1.264, *p* = 0.2559], lever [F (1, 152) = 2.160, *p* = 0.1437], and lever × day interaction [F (10, 152) = 0.4528, *p* = 0.9175]; post hoc analysis was performed using Bonferroni’s multiple comparisons test.

In contrast, cocaine-trained rats (‘cocaine’) exhibited a clear preference for the active lever associated with cocaine reinforcement, demonstrating the powerful, rewarding effects of the drug (Figure 1A). Two-way ANOVA with repeated measures of active lever presses over Days 1–11 revealed a significant effect of day [F (10, 198) = 1.998, *p* = 0.0353], lever [F (1, 198) = 162.9, *p* < 0.0001], and lever × day interaction [F (10, 198) = 2.648, *p* = 0.0047]; post hoc analysis was performed using Bonferroni’s multiple comparisons test. Importantly, when maintenance consumption of cocaine was attained, cocaine-trained rats displayed similar levels of drug consumption (Figure 1A).

#### 2.1.2. Iba-1 Levels Decreased in the Nucleus Accumbens

**Figure 2:**
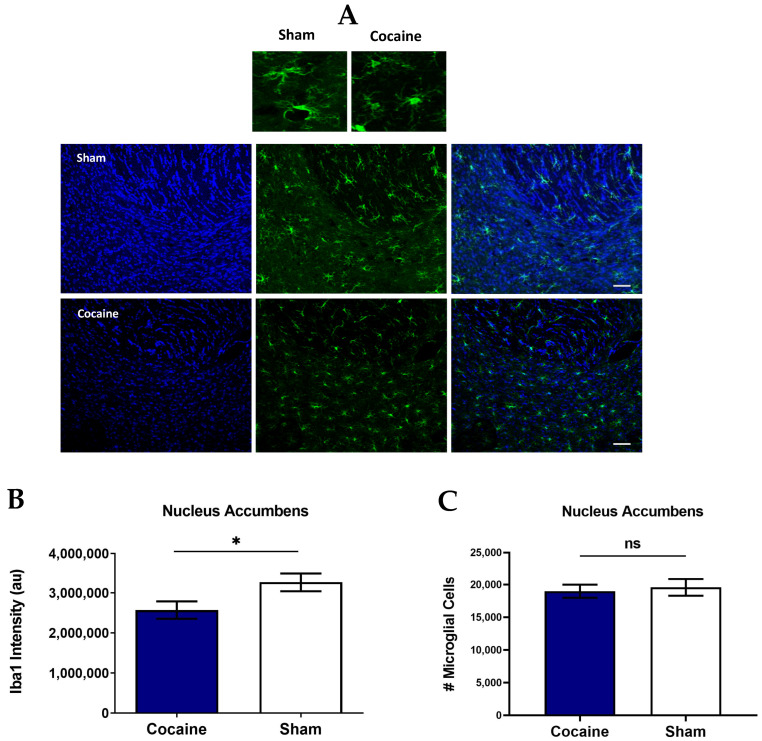
Cocaine self-administration impairs microglia in the Nucleus Accumbens; Iba-1 is decreased after reaching the maintenance dose of cocaine consumption. (**A**) Representative images showing Iba-1 in the Nucleus Accumbens (NAc) after cocaine maintenance consumption was attained. Scale bar: 25 μm. (**B**) Quantification of Iba-1 (au) in NAc. Iba-1 levels are significantly decreased in the cocaine-trained rats group compared to Sham. (**C**) Quantification of the number of Iba-1 + microglial cells in the NAc; cocaine maintenance consumption did not affect microglial cell number. Overall: * *p* < 0.05, no significant effect; ns two-tailed unpaired *t*-test, *n* = 8–9 per group.

Figure 2A presents representative Iba-1 staining confocal images of the NAc after reaching cocaine dose consumption (Figure 2A).

Unpaired *t*-test of Iba-1 levels showed a significant difference between groups: t(15) = 2.209, *p* = 0.0432. Cocaine-trained rats treated with the vehicle exhibited a significant decrease in Iba-1 levels compared with Sham rats (Figure 2B). Chronic cocaine consumption until reaching the maintenance stage consumption did not affect the number of microglial cells in the NAc: t(15) = 0.3732, *p* = 0.7143, quantified based on positive Iba-1 staining, but rather caused a decrease in the overall Iba-1 levels, suggesting impaired microglia rather than a reduction in cell numbers at this stage (Figure 2C). This decline in Iba-1 is not attributed to variations in drug consumption, as cocaine-trained rats exhibited similar intake based on a similar ratio in pressing the active lever vs. the demo during the maintenance phase.

#### 2.1.3. Iba-1 Levels Did Not Decrease in the Dentate Gyrus

**Figure 3:**
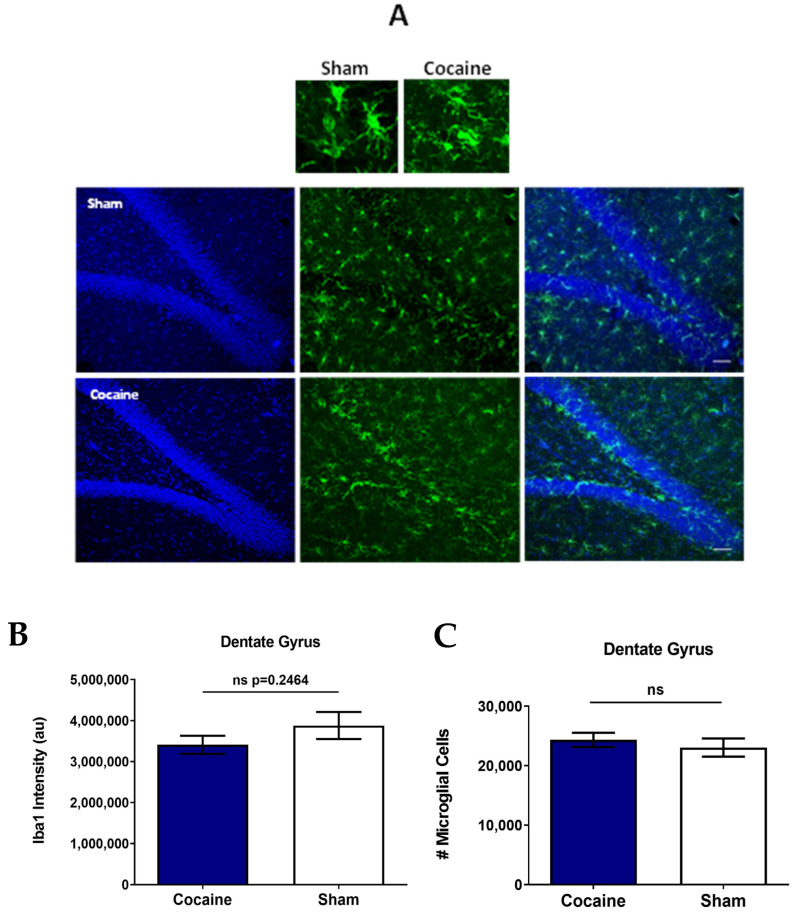
Cocaine self-administration does not impair microglia in the Dentate Gyrus after reaching the maintenance dose of cocaine consumption. (**A**) Representative images showing Iba-1 immunofluorescence in the Dentate Gyrus (DG) after cocaine maintenance consumption was attained. Scale bar: 25 μm. (**B**) Quantification of Iba-1 (au) in the DG. Iba-1 levels are not decreased in the cocaine-trained rats group compared to Sham. (**C**) Quantification of the number of Iba-1+ microglial cells in the DG; chronic consumption derived from cocaine self-administration and cocaine maintenance consumption did not affect microglial cell numbers. Overall: no significant effect; ns two-tailed unpaired *t*-test, *n* = 8–9 per group.

Figure 3A presents representative Iba-1 staining confocal images of the DG after reaching the cocaine dose consumption. An unpaired *t*-test of Iba-1 levels showed no significant difference between the groups: t(15) = 0.6626, *p* = 0.5176 (Figure 3B).

Also, chronic cocaine consumption until reaching the maintenance stage dose consumption stage did not affect the number of microglial cells in the DG: t(15) = 1.206, *p* = 0.2464 (Figure 3B), quantified based on positive Iba-1 staining and compared to Sham (Figure 3C), suggesting that the microglia are not impaired at this stage in the DG.

### 2.2. PLX-PAD Cells Attenuate Cocaine Craving in a Self-Administration Model

**Figure 4:**
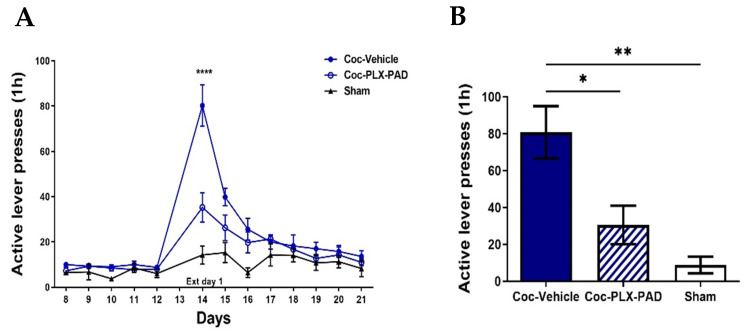
Effect of PLX-PAD therapy on long-term cocaine-seeking behavior; reinstatement test of usage behavior in a self-administration model. (**A**) Effect of cocaine-seeking behavior during extinction. On the first day of extinction (Day 14), the number of active lever presses was significantly higher in the cocaine group treated with vehicle (Plasma-Lyte), alleviated in the Coc-PLX-PAD group, and without any changes in the Sham group; two-way ANOVA with repeated measures and Bonferroni’s multiple comparison post hoc; **** *p* < 0.0001, *n* = 4–5. (**B**) Effect of IN PLX-PAD administration on reinstatement. The reinstatement test showed a significant effect of active lever presses in the Cocaine-PLX-PAD treatment compared to Cocaine-Vehicle on drug-seeking behavior. The Sham group did not demonstrate any change in the active lever presses in the reinstatement test; * *p* < 0.05, ** *p* < 0.01, *n* = 4–5, Tukey’s multiple comparisons test. Overall: data show mean ± SEM.

We examined the effect of PLX-PAD cell therapy on long-term cocaine-seeking behavior during the extinction and reinstatement to drug usage phases using three experimental groups: cocaine-trained rats treated with vehicle (‘cocaine’), PLX-PAD, and saline-trained rats (Sham) (Figure 4A). Two-way ANOVA with repeated measures of active lever presses over Days 1–21 in the three groups revealed significant effects of day [F (12, 125) = 24.62, *p* < 0.0001], group [F (2, 125) = 35.68, *p* < 0.0001], and group × day interaction [F (24, 125) = 7.110, *p* < 0.0001]; post hoc analysis was performed using Tukey’s multiple comparisons test. On Day 14, the first day of the extinction phase, cocaine-trained rats treated with vehicle exhibited significantly more (*p* < 0.0001) active lever presses than the PLX-PAD-treated groups, respectively, indicating higher craving for the drug. PLX-PAD-treated rats significantly pressed more (*p* < 0.001) on the active lever than the Sham group. On the next day (day 15), no significant difference was observed between PLX-PAD treatment and Sham groups.

Then, we assessed the rats’ propensity to relapse, assessed as cocaine-seeking behavior (active lever presses) after cocaine reinstatement on Day 22 (Figure 4B). One-way ANOVA of active lever presses on Day 22 showed a significant difference between groups [F(2,11) = 12.98, *p* = 0.0013]; post hoc analysis was performed using Tukey’s multiple comparison tests. Cocaine-trained rats treated with the vehicle exhibited significantly more (*p* < 0.05) active lever presses than the PLX-PAD-treated group. Dramatically, the number of active lever presses in cocaine-trained rats treated with PLX-PAD was not significantly different from that in Sham rats and was significantly lower than that in the corresponding vehicle-treated group (*p* < 0.01).

### 2.3. Iba-1 Levels After Cocaine Extinction Training and Reinstatement Test

To examine the effect of PLX-PAD cells on microglia, we compared the Iba-1 levels and the number of microglial cells in the NAc and DG of cocaine-trained rats treated with vehicle, PLX-PAD, and saline (Sham) after extinction training and the reinstatement test. Moreover, to support the biological findings with behavioral results, we examined the correlation between active lever presses and Iba-1 levels.

#### 2.3.1. Iba-1 Levels Decreased in the Nucleus Accumbens

**Figure 5:**
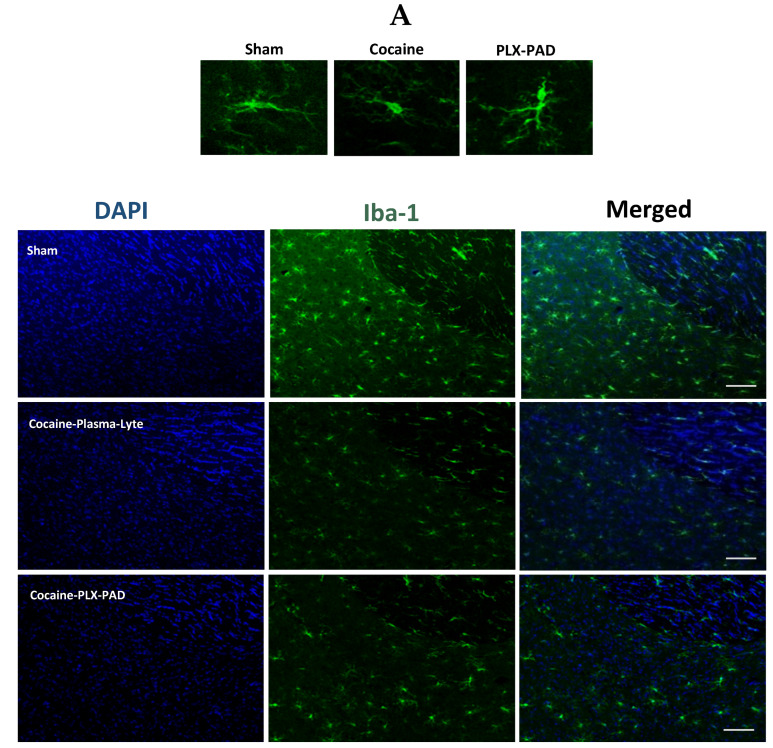

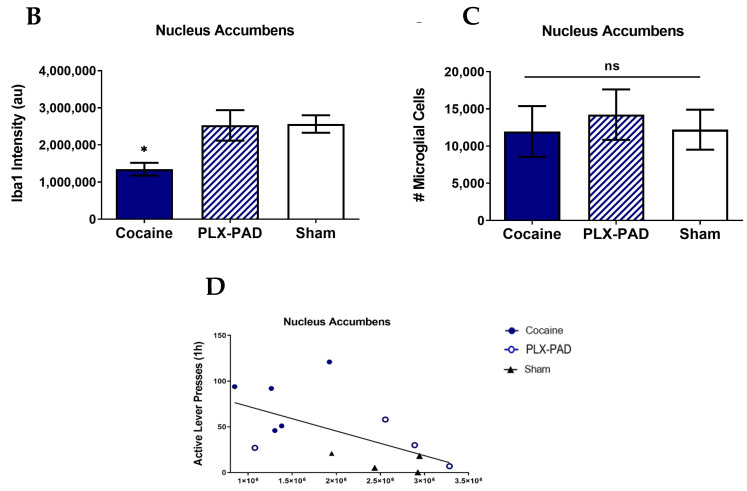
Cocaine self-administration impairs microglia in the Nucleus Accumbens. Iba-1 decreased after the extinction training and reinstatement test. (**A**) Representative images showing Iba-1 immunofluorescence in the Nucleus Accumbens (NAc) after saline or cocaine extinction training and reinstatement test. Scale bar: 75 μm. (**B**) Quantification of Iba-1 (au) in the NAc. Iba-1 levels are significantly decreased in cocaine-Plasma-Lyte compared to Sham, and the PLX-PAD cell therapy restores to corresponding levels as Sham. (**C**) Quantification of the number of Iba-1+ microglial cells in the NAc; chronic cocaine-derived cocaine self-administration followed by extinction training and the reinstatement test did not affect microglial cell number. Overall: * *p* < 0.05, no significant effect; ns, Tukey’s multiple comparisons, *n* = 4–5 per group. (**D**) Correlation between drug-seeking behavior in the reinstatement test (Figure 4B and Iba-1 (au) (**B**)). Scatter plot shows Pearson correlation coefficient r = −0.5848, * *p* = 0.0358, *n* = 4–5 per group. Overall: data show mean ± SEM.

Representative Iba-1 staining confocal images of the NAc (Figure 5A) show a decrease in Iba-1 levels after cocaine extinction training followed by the reinstatement test in the cocaine-trained rats (cocaine) compared with Sham (saline) rats. One-way ANOVA of total Iba-1 levels in the NAc showed a significant difference between groups [F (2, 10) = 6.750, *p* = 0.0140]; post hoc analysis was performed using Tukey’s multiple comparisons test. The vehicle-treated cocaine-trained group exhibited a decrease in the Iba-1 levels compared to the saline-trained (Sham) and PLX-PAD treatment groups (*p* < 0.05). In rats treated with PLX-PAD cells, Iba-1 levels were as high as in the Sham group (Figure 5B), indicating that PLX-PAD cells prevented the decrease in Iba-1 levels affecting the microglia functioning.

Chronic cocaine exposure did not affect the number of microglial cells in the NAc, quantified based on positive Iba-1 staining, but rather caused a decrease in the overall Iba-1 levels (Figure 5C), indicating it impaired microglia activity rather than reduced their numbers. To determine whether the restoration of microglia impairment correlated with decreased cocaine craving, we correlated the number of active lever presses during the relapse test (Figure 4B) with Iba-1 levels observed in the same rats (Figure 5B). A statistically significant correlation was found (Pearson correlation coefficient r = −0.5848, *p* = 0.0358), with a distinct division between the Sham and the PLX-PAD cell treatment groups and the cocaine-trained rats treated with vehicle. This suggests that microglia may have contributed to the decreased cocaine craving. Therefore, IN PLX-PAD treatment was able to rescue the microglia impairment caused by cocaine training and attenuate cocaine craving.

Hence, although cocaine training impaired the microglia in the NAc during drug consumption and withdrawal, PLX-PAD cell treatment restored the microglia and attenuated cocaine craving (Figure 5D).

#### 2.3.2. Iba-1 Levels Decreased in the Dentate Gyrus

**Figure 6:**
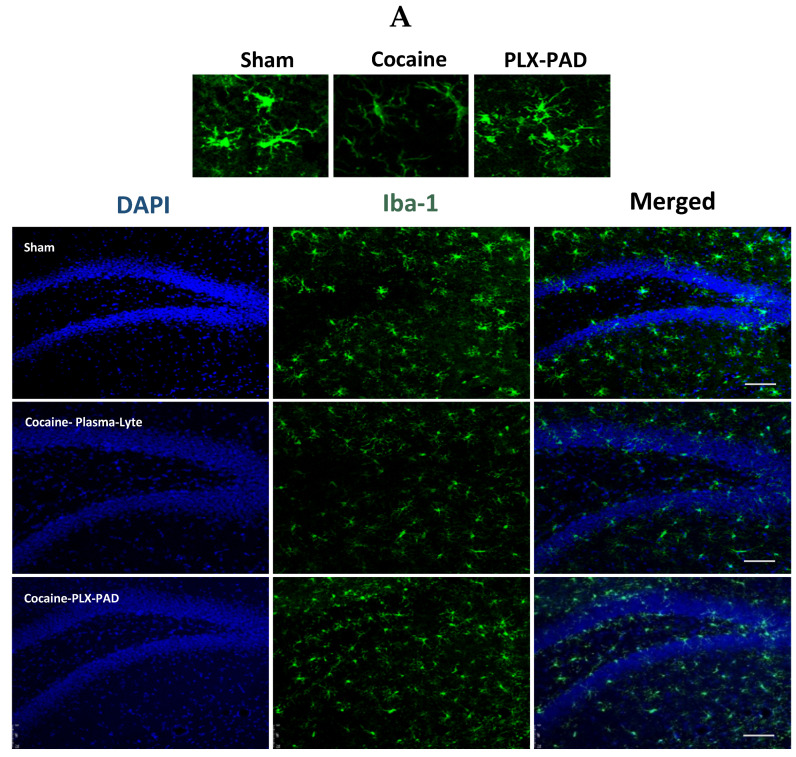

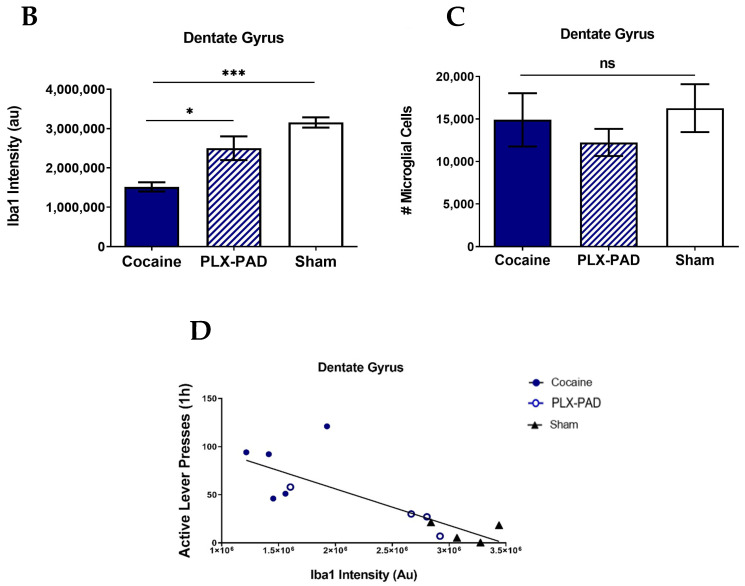
Cocaine self-administration impairs microglia in the Dentate Gyrus. Iba-1 level is decreased after extinction training and the reinstatement test. (**A**) Representative images showing Iba-1 immunofluorescence in the Dentate Gyrus (DG) after Sham or cocaine extinction training and the reinstatement test. Scale bar: 75 μm. (**B**) Quantification of Iba-1 (au) in DG. Iba-1 levels are significantly decreased in cocaine-Plasma-Lyte compared to Sham, and the PLX-PAD cell therapy restores Iba-1 levels to corresponding levels as Sham. (**C**) Quantification of the number of Iba-1+ microglial cells in the DG; chronic cocaine use derived from cocaine self-administration, followed by extinction training, and the reinstatement test did not affect microglial cell number. Overall: * *p* < 0.05, *** *p* < 0.001, no significant effect; ns, Tukey’s multiple comparisons, *n* = 4–5 per group. (**D**) Correlation between drug-seeking behavior in the reinstatement test (Figure 4B) and Iba-1 (au) (**B**). Scatter plot shows Pearson correlation coefficient r = −0.5848, * *p* = 0.0358, *n* = 4–5 per group. Overall: Data show mean ± SEM.

Similar results were observed in the Dentate Gyrus. Representative Iba-1 staining confocal images of the DG (Figure 6A) show a decrease in Iba-1 levels after cocaine extinction training, followed by the reinstatement test in the cocaine-trained rats (cocaine) compared with Sham rats. One-way ANOVA of total Iba-1 levels in the DG showed a significant difference between groups [F (2, 10) = 19.60, *p* = 0.0003]; post hoc analysis was performed using Tukey’s multiple comparisons test.

The vehicle-treated cocaine-trained group exhibited a decrease in the Iba-1 levels compared to the saline-trained (Sham) and PLX-PAD treatment groups (*p* < 0.001 and *p* < 0.05, respectively). In rats treated with PLX-PAD cells, Iba-1 levels were as high as those observed in the Sham group and in the same magnitude (Figure 6B), indicating that PLX-PAD cells prevented the decrease in Iba-1 levels and its impact on the microglia. Chronic cocaine exposure did not affect the number of microglial cells in the DG, as quantified based on positive Iba-1 staining, but rather caused a decrease in the overall Iba-1 levels (Figure 6C), indicating it impairs microglia activity rather than reduces their numbers.

To determine whether the restoration of microglia impairment correlated with a decrease in cocaine craving, we correlated the number of active lever presses during the relapse test (Figure 4B) with Iba-1 levels observed in the same rats (Figure 6B). A statistically significant correlation was found (Pearson correlation coefficient r = −0.7976, *p* = 0.0011), with a distinct division between the Sham and the PLX-PAD cell treatment groups and the cocaine-trained rats treated with vehicle. This suggests that the restored microglia activity may have contributed to the decreased cocaine craving. Therefore, IN PLX-PAD treatment was able to rescue the microglia impairment caused by cocaine training and attenuate cocaine craving.

Hence, although cocaine training impaired the microglia in the DG during drug consumption and withdrawal, PLX-PAD cell treatment restored the microglia and attenuated cocaine craving (Figure 6D).

### 2.4. Soma Area of Microglial Cells in the Dentate Gyrus and the Nucleus Accumbens by Groups and Treatments

Changes in microglial morphology could indicate a microglial activation state [54]. To support the biological findings, we assessed the cell body area and branches of the microglial cells in the NAc and DG in all treatment stages, comparing cocaine-trained rats treated with vehicle, PLX-PAD, and saline (Sham).

#### 2.4.1. Soma Area and Branches in the Nucleus Accumbens

**Figure 7:**
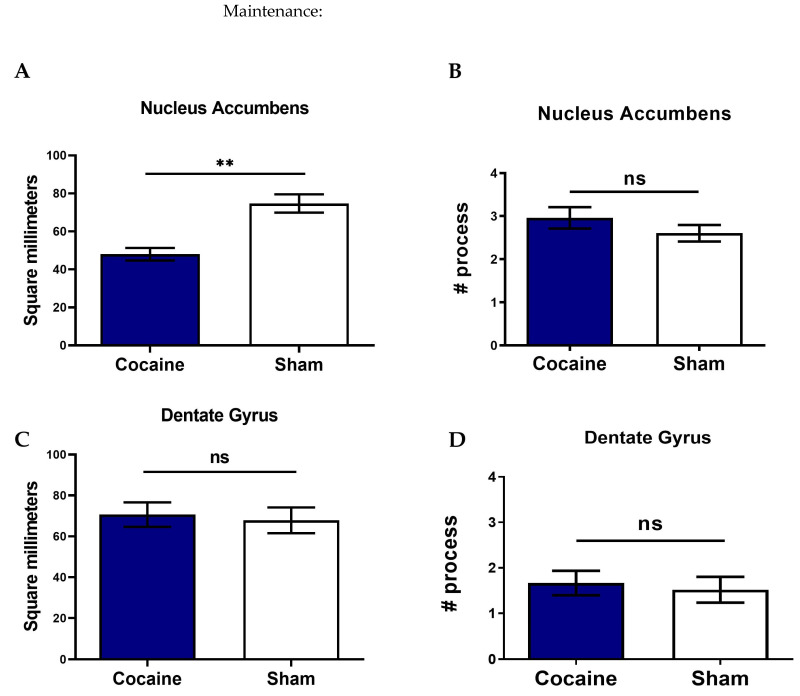

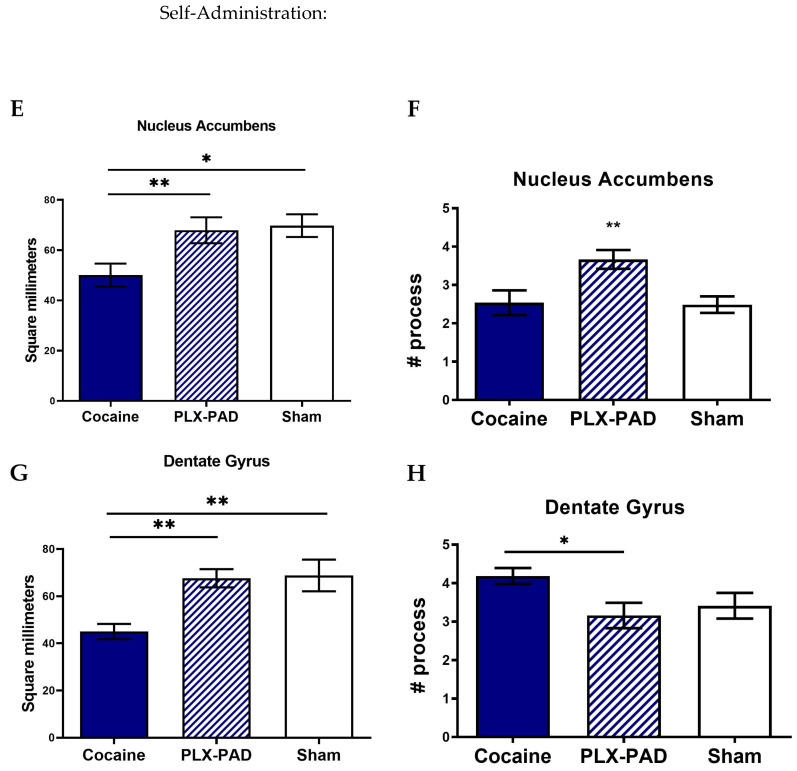
Comparisons of cell body area (soma) and branches of microglial cells between the different treatments in the Dentate Gyrus (DG) and Nucleus Accumbens (NAc) tissues during the maintenance and self-administration stages. (**A**) Assessment of the microglial soma area in the NAc in the maintenance stage. The soma area is significantly decreased in the cocaine-trained rats compared to in Sham. (**B**) Assessment of the microglial branches in the NAc in the maintenance stage. There is no difference between cocaine-trained rats and Sham. (**C**) Assessment of the microglial soma area in the DG in the maintenance stage. There is no difference between cocaine-trained rats and Sham. (**D**) Assessment of the microglial branches in the in the DG in the maintenance stage. There is no difference between cocaine-trained rats and Sham. (**E**) Assessment of the microglial soma area in the NAc during the self-administration stages. The soma area is significantly decreased in the cocaine-trained rats treated with vehicle compared to cocaine-trained rats treated with PLX-PAD and Sham, but not between PLX-PAD-treated rats and Sham. (**F**) Assessment of the microglial branches in the NAc during the self-administration stages. There are significantly increased branches in the PLX-PAD- treated rats compared to cocaine-trained rats treated with vehicle and Sham, but not between cocaine-trained rats treated with vehicle and Sham. (**G**) Assessment of the microglial soma area in the DG during the self-administration stages. The soma area is significantly decreased in the cocaine-trained rats treated with vehicle compared to PLX-PAD- treated rats and Sham, but not between PLX-PAD- treated rats and Sham. (**H**) Assessment of the microglial branches in the DG during the self-administration stages. There are significantly increased branches in the cocaine-trained rats treated with vehicle compared to PLX-PAD- treated rats and Sham, but not between PLX-PAD- treated rats and Sham. Overall: * *p* < 0.05, ** *p* < 0.01, no significant effect; ns, Tukey’s multiple comparisons. Overall: data show mean ± SEM.

Image analysis showed that in the maintenance phase, the soma area decreased in the NAc (*p* < 0. 01) (Figure 7A), with no change in the branches (Figure 7B) in the cocaine-trained rats treated with vehicle group compared to Sham. In the self-administration stages, a decreased soma area was found in the cocaine-trained rats treated with vehicle compared to PLX-PAD-treated rats (*p* < 0.01) and Sham (*p* < 0.05) (Figure 7E), while an increase in the branches was shown only in the PLX-PAD-treated rats (*p* < 0.01) (Figure 7F).

#### 2.4.2. Soma Area and Branches in the Dentate Gyrus

Image analysis showed that in the maintenance phase, the soma area did not decrease in the DG (Figure 7C), with no change in the branches (Figure 7D) in the cocaine-trained rats treated with vehicle group compared to Sham. In the self-administration stages, a decreased soma area was found in the cocaine-trained rats treated with vehicle compared to PLX-PAD-treated rats (*p* < 0.01) and Sham (*p* < 0.01) in the DG (Figure 7G), while an increase in the branches was shown only in the cocaine-trained rats treated with vehicle compared to PLX-PAD-treated rats (*p* < 0.05) (Figure 7H).

Table 1 summarizes the results of the biological (Iba-1), morphological (soma area and branches), and behavioral (lever presses) assessments in the cocaine-trained rats treated with vehicle, PLX-PAD, and Sham in all the self-administration stages.

## 3. Discussion

This study aimed to uncover the relationship between microglia and cocaine cravings and investigate whether PLX-PAD can modulate this effect using the self-administration model. We focused on two brain regions, the Nucleus Accumbens and Dentate Gyrus, assessing microglial impairment by measuring Iba-1 levels, the number of Iba-1 + labeled cells, and the microglial cell morphology while paralleling these findings with cocaine craving as measured by active lever presses.

The results demonstrated that cocaine impaired microglial function in a stage- and region-specific manner. Decreased Iba-1 levels and soma area of microglia were observed in the NAc but not in the DG after the achievement of the maintenance dose of cocaine consumption. This aligns with studies indicating that the NAc plays a critical role in switching from drug use to drug abuse and compulsive drug-seeking behavior, which strengthens cocaine craving [52]. The decreased Iba-1 levels found in this study may seem in contrast to studies showing an increase in the maintenance stage [36,44]. However, if the decreased levels result from the excessive activation of microglia leading to microglia dysfunction, the contrast disappears. The difference between the increase and decrease in expression is likely due to a dose-dependent activation threshold. The dose used in this research was known to impair neurogenesis [31,32], probably leading to greater involvement of microglia and resulting in impairment, likely due to excessive activation [37].

Furthermore, microglial deficiencies were evidenced by decreased Iba-1 levels and soma in both NAc and DG after the extinction training and reinstatement tests. Notably, ramified microglia were observed only after the extinction reinstatement in the PLX-PAD-treated rats in the NAc and vehicle-treated rats in the DG. These findings support studies indicating that the hippocampus, which is associated with neurogenesis and learning and memory, is strongly involved during the later stages of cocaine addiction, such as withdrawal and relapse [53], and indicating the involvement of microglia in modulating cocaine addiction behaviors [55]. The results suggest that learning and memory, as well as forgetting, are driven by natural microglia activity. Once this process is impaired by cocaine, forgetting to be addicted can be achieved through PLX-PAD treatment [5]. This observation is consistent with a previous study showing that Iba-1 decreased in the NAc after extinction training in the cocaine self-administration mice model [44]. The results also agree with studies showing that the plasticity changes caused by chronic cocaine exposure may lead to microglial dysfunction [55], as microglia maintain neuronal health and regulate adult neurogenesis [2,56]. Microglia remove unnecessary synaptic input, modulate neural circuits [8,9], and secrete factors that control neurogenesis [10,56]. Therefore, neurogenesis and neural connectivity, which are important components of the rehabilitation process, rely on the proper functioning of microglia.

Interestingly, the number of microglial cells remained intact across conditions. Since Iba-1 is expressed by both reactive and quiescent microglial cells [33,35], the observed decline in Iba-1 immunoreactivity likely reflects a microglia impairment rather than necrosis or cell death. Recent studies highlight that the elimination of the microglia-specific protein Iba-1, a unique marker for microglia whose role remains unclear, leads to structural and functional impairments in microglia. These impairments have a notable impact on both synaptic development and behavior. Furthermore, it was established that Iba-1 is pivotal in directing microglial activity during essential neuroglial developmental processes, potentially having a profound influence on behavior [57]. While our study did not directly measure the outcome of microglial activity, fluctuations in Iba-1 levels were interpreted as indicative of microglia impairment, correlating with synaptic and behavioral disruptions.

Our findings are supported by studies showing that microglia in various regions have different sensitivities to cocaine exposure, as well at the stages of withdrawal and relapse [36,44]. More support is found in research revealing that microglia consist of a heterogeneous population of cells in various brain regions, possessing distinct properties and functional specializations [58]. Upon exposure to environmental stimuli, microglial subtypes may respond by altering their biology and gene expression, leading to the manifestation of a specific phenotype [59]. Further investigation is needed to expand this research, including a comprehensive exploration of other limbic brain regions, such as the ventral tegmental area (VTA), which mediates elevated synaptic dopamine levels in the NAc and the hippocampus that lead to reward [60].

The findings also show that PLX-PAD significantly decreased cocaine craving and cocaine-seeking behavior during extinction, mainly at reinstatement, over the long term. Notably, the treatment with PLX-PAD cells prevented the decrease in Iba-1 levels and restored them to those observed in the Sham group, reinforcing that cocaine use impairs the microglia and microglial involvement in addiction. Additionally, the rehabilitation effects corresponded to brain tissue regeneration: the restoration of neurogenesis in the DG and repair of microglial functioning in the NAc and DG, which enabled forgetting the compulsive cocaine reward. This coincides with MSCs having immunomodulatory properties and promoting neural cell survival and regeneration (neurogenesis, angiogenesis, and synaptogenesis) [54]. It is also supported by a study on neonatal hypoxic-ischemic brain injury in mice that shows that MSC treatment increased neurogenesis and modulated microglial activation [23]. Moreover, microglia were reported as a main factor in shaping the spines of new neurons [5,13,14,15]. As such, this study shows the need for microglia to link neurogenesis to functional connectivity, leading to restoration and the alleviation of cocaine-seeking behavior [32]. This study uncovers essential microglial function by applying neurogenesis to the connectivity function, providing insights into potential avenues for addiction treatment.

To conclude, this study reveals that microglial dysfunction in the NAc and DG is stage- and region-dependent along the progression of cocaine addiction. Also, it highlights the powerful therapeutic potential of PLX-PAD cells in attenuating cocaine-seeking behavior by targeting mesolimbic regions and thereby improving their plasticity by restoring neurons in the hippocampus and repairing microglial functioning activity, which, in turn, promotes neuroplasticity and neurogenesis. Moreover, it highlights the critical role of microglia in synaptic plasticity and neuronal remodeling associated with memory formation, underscoring the necessity of considering microglia in the comprehensive treatment of addiction rehabilitation. The use of intranasal administration for PLX-PAD cells adds significant value to this study. Intranasal delivery replicates the pharmacokinetics, pharmacodynamics, and behavioral effects of intracerebroventricular administration while being less invasive and minimizing the risks of contamination and brain tissue damage. Furthermore, intranasally administered PLX-PAD cells can cross the blood–brain barrier and selectively target addiction-related brain regions, delivering their therapeutic “cargo” locally while minimizing systemic side effects. This approach not only reduces pain in animal experiments but also enhances the feasibility of clinical application.

## 4. Materials and Methods

### 4.1. Subjects

Male Sprague Dawley rats (250–280 g) were used. Rats were purchased from Envigo Inc. (Indianapolis, IN, USA) and maintained (in pairs) on a 12–12 h light–dark cycle (lights off at 07:00 a.m.) with free access to food and water. All experimental procedures were approved by the Animal Care and Use Committee of Bar Ilan University and performed in accordance with the guidelines of the National Institute of Health.

### 4.2. Intravenous Catheterization

Rats were anesthetized with ketamine hydrochloride [100 mg/kg, intraperitoneally (i.p.)] and xylazine (10 mg/kg, i.p.), then implanted with intravenous Silastic catheters (Dow Corning, Midland, MI, USA) into the right jugular vein. The catheter was secured to the vein with silk sutures and was passed subcutaneously to the top of the skull, where it exited into a connector (a modified 22-gauge cannula, Plastics One, Roanoke, VA, USA) that was mounted to the skull with MX-80 screws (Small Parts, Inc., Miami Lakes, FL, USA) and dental cement (Yates and Bird, Chicago, IL, USA). Catheters were used for self-administration of cocaine or saline.

### 4.3. Cocaine Self-Administration

Rats were trained to self-administer cocaine (cocaine was obtained from the National Institutes of Drug Abuse, North Bethesda, MD, USA) as previously described [32] under an FR-1 schedule of reinforcement for 11–13 days until reaching stable maintenance of drug intake, as follows: Five days after catheterization, rats were transferred to operant conditioning chambers (Med-Associates, Inc., St. Albans, VT, USA) for one hour daily during their dark cycle. Each self-administration chamber (30 × 25 × 22 cm) had two levers, active and inactive, located 9 cm above the chamber’s floor. The self-administration chambers and the computer interface were built locally and controlled by a computer program written by Steve Cabilio (Concordia University, Montreal, PQ, Canada; steve.cabilio@concordia.ca). An active lever press generated a cocaine infusion (1.5 mg/kg in saline, 0.13 mL total volume, over 20 s) through the IV catheter connected to an infusion pump. During cocaine infusion, a light located above the active lever was lit for 20 s. During the 20 s infusions, active lever presses were recorded, but no additional cocaine reinforcement was provided. Presses on the inactive lever did not activate the infusion pump or light. The number of active lever presses, infusions, and inactive lever presses was recorded. Rats were returned to their home cages at the end of the daily session. Sham groups underwent the same self-administration protocol with saline instead of cocaine. PLX-PAD or vehicle (or gold nanoparticles in navigation experiments, below) was administered intranasally or intracerebroventricularly 24 h after the last self-administration session.

### 4.4. Cocaine Extinction and Reinstatement to Cocaine

During the extinction period, rats were placed in the operant chamber for 60 min daily sessions with no cocaine or saline access (active and inactive lever presses were recorded but had no effect). Rats underwent drug extinction training for 10 days until the number of active lever presses decreased significantly from the first day of drug extinction training and was, therefore, assumed to be non-reinforced by cocaine. The rats used in navigation experiments were sacrificed during the extinction period.

The reinstatement test to assess cocaine craving during relapse was performed 24 h after completion of the last extinction session (or 28 days after BrdU injection in neurogenesis experiments), as follows: Cocaine-trained rats were primed with a single cocaine injection (10 mg/kg i.p.), then placed in the self-administration (operant) chamber for 1 h, with no cocaine dispensed; lever presses were monitored but had no effect. Sham groups underwent the same relapse protocol with saline instead of cocaine. Rats completing the relapse test were decapitated, and their brains were removed.

### 4.5. Intranasal Administration

Rats that were to undergo intranasal (IN) administration were anesthetized either by i.p. injection of 100 mg/kg ketamine and 10 mg/kg xylazine or by placement for 20 min into an anesthesia chamber the size of the normal home cage containing clean bedding with 2% isoflurane and 98% air. Then, 1 × 10^6^ PLX-PAD cells in 50 µL within 1 h from thawing (or vehicle: 50 µL PlasmaLyte) were administered IN using the Impel Rat Intranasal Catheter Device (Neuropharma, Israel) using SP2 settings, half into each nostril. The catheter tube was inserted into the nostril (5 mm deep) using a guide catheter. Cells were slowly released inside each nostril (25 µL/15 min), ensuring they did not touch the nasal mucus. To enable a maximum number of cells to pass without leaking, the rat’s head was held tilted up at 15 degrees.

### 4.6. Microglial Iba-1 Assessment

The experiment was conducted as described in the Methods—using a cocaine dose known to impair neurogenesis [31,32], the same dose used in all experiments in this study. Briefly, rats underwent cocaine or saline training in the self-administration model. At the conclusion of the experiment, the rats were sacrificed and perfused, and their brains were removed. The DG and NAc slices were collected and stained with Iba-1, a marker for functioning microglia, and DAPI, *n* = 4–5.

#### 4.6.1. Microglial Iba-1 Staining

40 μm coronal brain sections (8 sections for NAc and 10 sections for DG) were rinsed three times with PBS, then treated with blocking buffer (Triton X-100% 0.1 and PBS containing serum horse normal 20%) for 1 h. The primary antibody, rabbit anti-Iba-1 (1:1000; Wako, Osaka, Japan), was incubated overnight at 4 °C. After washing slides three times in PBS, the secondary antibody, Alexa Fluor488 goat anti-rabbit (1:200; Invitrogen, Waltham, MA, USA), was added for 1 h at room temperature. The slides were rinsed three times with a solution containing PBS and Triton X-100% 0.1, followed by staining with DAPI (1:1000) for 10 min to visualize cell nuclei, and then were rinsed three times with a solution containing PBS and Triton X-100% 0.1.

#### 4.6.2. Iba-1 Immunohistochemistry Analysis

Confocal brain images were taken at 20× magnification (15–20 µm z-stack with 2 µm step) using Leica inverted SP8 scanning confocal microscope (Leica Microsystems, Mannheim, Germany) using LASX software (version 3.5.2.18963). The 3D stack was max projected into a 2D image using LASX. For the calculation of the Iba-1 immunoreactivity, images were then exported as TIF files and imported into CellProfiler (version 3.0). Using the green channel, cells were segmented, and integrated intensity for every cell was measured. Values were exported to Excel and then summed per image, then per rat, and then averaged between rats from each group. The final value is intensity-integrated staining expressed in AU. The CellProfiler pipeline is available upon request.

### 4.7. Microscope

All brain images were taken using a Leica SP8 Leica inverted SP8 scanning confocal microscope driven by the LASX software and using an HC PL APO 20×/0.75 objective (Leica Microsystems, Mannheim, Germany) at 20× magnification to produce multiple z-stack images for further analysis. To minimize batch effects during the staining process, tissue slices were randomly selected from all experimental groups. Also, all staining procedures were carried out on consecutive days, ensuring that environmental factors, reagent handling, and other technical variables remained consistent and maintained equal conditions across the staining procedure. Moreover, all slices were processed using identical reagents to maintain consistency throughout the staining protocol. Furthermore, imaging sessions were conducted on randomized slices from all groups each day, using consistent settings to minimize potential batch effects.

### 4.8. Image Analysis

To assess the cell body area of microglial cells, images were acquired using a Leica SP8 inverted scanning confocal microscope equipped with LASX software. Imaging was conducted in two regions: the Dentate Gyrus (DG) and the nucleus accumbens (NAc). Cell body areas were quantified using ImageJ/FIJI 1 software with the Wand tool. Cells with cell body areas that were statistical outliers relative to the distribution of other cells within the same image were excluded from the analysis.

### 4.9. Statistical Analysis

All data are expressed as mean ± SD. Unpaired *t*-test and one or two-way ANOVA with repeated measures (days), followed by Tukey’s or Bonferroni’s multiple comparison tests, were used as appropriate. Pearson’s or Spearman’s tests were used to analyze continuous variables. Results were considered significantly different if *p* < 0.05. All the data were analyzed using Prism 8 software (GraphPad, San Diego, CA, USA).

## 5. Patents

The patent with Pluristem is https://patentscope.wipo.int/search/en/detail.jsf?docId=WO2019021158&_cid=P10-L0GOQ9-64402-1 (accessed on 5 May 2022).

## Figures and Tables

**Table 1:** A summary of the results in all self-administration stages.

	Maintenance		Extinction Training and Reinstatement			
	Cocaine-Trained Rats Treated Vehicle		Cocaine-Trained Rats Treated Vehicle		Cocaine-Trained Rats Treated PLX-PAD	
	Nucleus Accumbens	Dentate Gyrus	Nucleus Accumbens	Dentate Gyrus	Nucleus Accumbens	Dentate Gyrus
#Cells	unchanged	unchanged	unchanged	unchanged	unchanged	unchanged
Iba-1	decrease	unchanged	decrease	decrease	like Sham	like Sham
Soma area	decrease	unchanged	decrease	decrease	like Sham	like Sham
Branches	unchanged	unchanged	decrease	increase	increase	like Sham
Lever presses	-	-	increase		like Sham	

## Data Availability

The raw data supporting the conclusions of this article will be made available by the authors upon request.
